# WhatsApp Messenger as an Adjunctive Tool for Telemedicine: An Overview

**DOI:** 10.2196/ijmr.6214

**Published:** 2017-07-21

**Authors:** Vincenzo Giordano, Hilton Koch, Alexandre Godoy-Santos, William Dias Belangero, Robinson Esteves Santos Pires, Pedro Labronici

**Affiliations:** ^1^ Department of Orthopedics Hospital Municipal Miguel Couto Rio de Janeiro Brazil; ^2^ Department of Radiology Universidade Federal do Rio de Janeiro Rio de Janeiro Brazil; ^3^ Department of Orthopedics Universidade Federal de São Paulo São Paulo Brazil; ^4^ Department of Orthopedics Universidade Estadual de Campinas Campinas Brazil; ^5^ Department of Orthopedics Universidade Federal de Minas Gerais Belo Horizonte Brazil; ^6^ Department of Orthopedics Universidade Federal Fluminense Niterói Brazil

**Keywords:** whatsapp, telemedicine, smartphone, mobile application

## Abstract

**Background:**

The advent of telemedicine has allowed physicians to deliver medical treatment to patients from a distance. Mobile apps such as WhatsApp Messenger, an instant messaging service, came as a novel concept in all fields of social life, including medicine. The use of instant messaging services has been shown to improve communication within medical teams by providing means for quick teleconsultation, information sharing, and starting treatment as soon as possible.

**Objective:**

The aim of this study was to perform a comprehensive systematic review of present literature on the use of the WhatsApp Messenger app as an adjunctive health care tool for medical doctors.

**Methods:**

Searches were performed in PubMed, EMBASE, and the Cochrane Library using the term “whatsapp*” in articles published before January 2016. A bibliography of all relevant original articles that used the WhatsApp Messenger app was created. The level of evidence of each study was determined according to the Oxford Levels of Evidence ranking system produced by the Oxford Centre for Evidence-Based Medicine. The impact and the indications of WhatsApp Messenger are discussed in order to understand the extent to which this app currently functions as an adjunctive tool for telemedicine.

**Results:**

The database search identified a total of 30 studies in which the term “whatsapp*” was used. Each article’s list of references was evaluated item-by-item. After literature reviews, letters to the editor, and low-quality studies were excluded, a total of 10 studies were found to be eligible for inclusion. Of these studies, 9 had been published in the English language and 1 had been published in Spanish. Five were published by medical doctors.

**Conclusions:**

The pooled data presents compelling evidence that the WhatsApp Messenger app is a promising system, whether used as a communication tool between health care professionals, as a means of communication between health care professionals and the general public, or as a learning tool for providing health care information to professionals or to the general population. However, high-quality and properly evaluated research is needed, as are improvements in descriptions of the methodology and the study processes. These improvements will allow WhatsApp Messenger to be categorically defined as an effective telemedicine tool in many different fields of health care.

## Introduction

Telemedicine is defined as the use of electronic information and communication technologies to provide health care support when distance separates the client (the patient or health care worker) from the health care professional with expertise in the relevant field [1,2]. The information transmitted between the two parties can take many forms including data and text, audio, still images, and video [3].

The use of mobile technologies in general and mobile phone specifically, is a rapidly expanding field within telemedicine [4]. Audiovisual communication in health care aided by smartphone apps is a novel concept that is rapidly gaining ground in all areas of medicine [5]. A nationwide survey performed in the United States in 2011 included 3306 medical providers and found that more than half used various apps in their clinical practice, some of which had not been specifically developed for medical purposes [6].

Currently, one of the most popular nonmedical mobile apps is WhatsApp Messenger, which has been downloaded in 40 countries in Europe, Asia, the Middle East, and the Americas. After reaching 1 million users at the end of 2009, WhatsApp Messenger app downloads increased tenfold in 2010 [7]. WhatsApp Messenger is a communication tool that allows users to send instant messages, photos, video, and voice messages and to make voice calls over an Internet connection [8]. Its main feature is to help people stay connected by sending and receiving messages at no per-message cost to the user (in contrast to original texting [short message service, SMS] services on mobile phones). The requirement of only a mobile Internet connection (whether via a data plan or Wi-Fi) explains the app’s widespread success [5].

Although scientific studies on the use of WhatsApp Messenger remain scarce in the medical literature, increasing numbers of health professionals have adopted it as a communication interface and for the exchange of images and videos [9,10]. Its use does not seem to reduce image quality in the conversion from analog to digital formats, thus providing the ability to identify sufficient details for an adequate diagnosis and initial treatment with better efficacy than other modalities used for the same purposes [11-13].

The aim of this study was to perform a comprehensive systematic review of current literature on the use of the WhatsApp Messenger mobile app as an adjunctive health care tool, as there is some evidence that this app can be an effective, safe, and economical telemedicine tool for professionals from all fields of health care (nurses, psychologists, dentists, physical therapists, and physical and sports educators, among others).

## Methods

### Search Strategy and Study Selection

A systematic electronic search of the PubMed, EMBASE, and the Cochrane Library databases was performed to find all literature using the term “WhatsApp[All fields]” and published before January 2016.

The inclusion criteria included (1) an evaluation of the impact of WhatsApp Messenger as one of the primary outcome measurements, (2) a conclusion containing clear indications for the use of the app, and (3) adult patients over the age of 18 years. The exclusion criteria included (1) case reports or case series with fewer than 5 patients, (2) other reviews of the literature, (3) meta-analyses, and (4) letters to the editor. No study was excluded due to the original language in which it was written.

### Data Extraction

The main author of this study (VG) evaluated all of the articles and applied the inclusion and exclusion criteria. Each relevant study was obtained and reviewed in its entirety. The level of evidence was determined according to the Oxford Levels of Evidence ranking system produced by the Oxford Centre for Evidence-Based Medicine [14].

The basic information about the article was extracted from the eligible articles. This basic information included the original language, the country where the research was performed, the type of participants (medical professionals, other health care professionals, or the general population), the medical specialty considered, the number of participants in the study, mean participant age, the results, and any problems observed.

### Sensitivity Analysis

A sensitivity analysis was performed to determine the robustness and reliability of the results of this study. Literature reviews, letters to the editor, and low-quality studies were excluded from the final analysis.

## Results

### Literature Search Strategy and Quality Assessment

**Figure 1:**
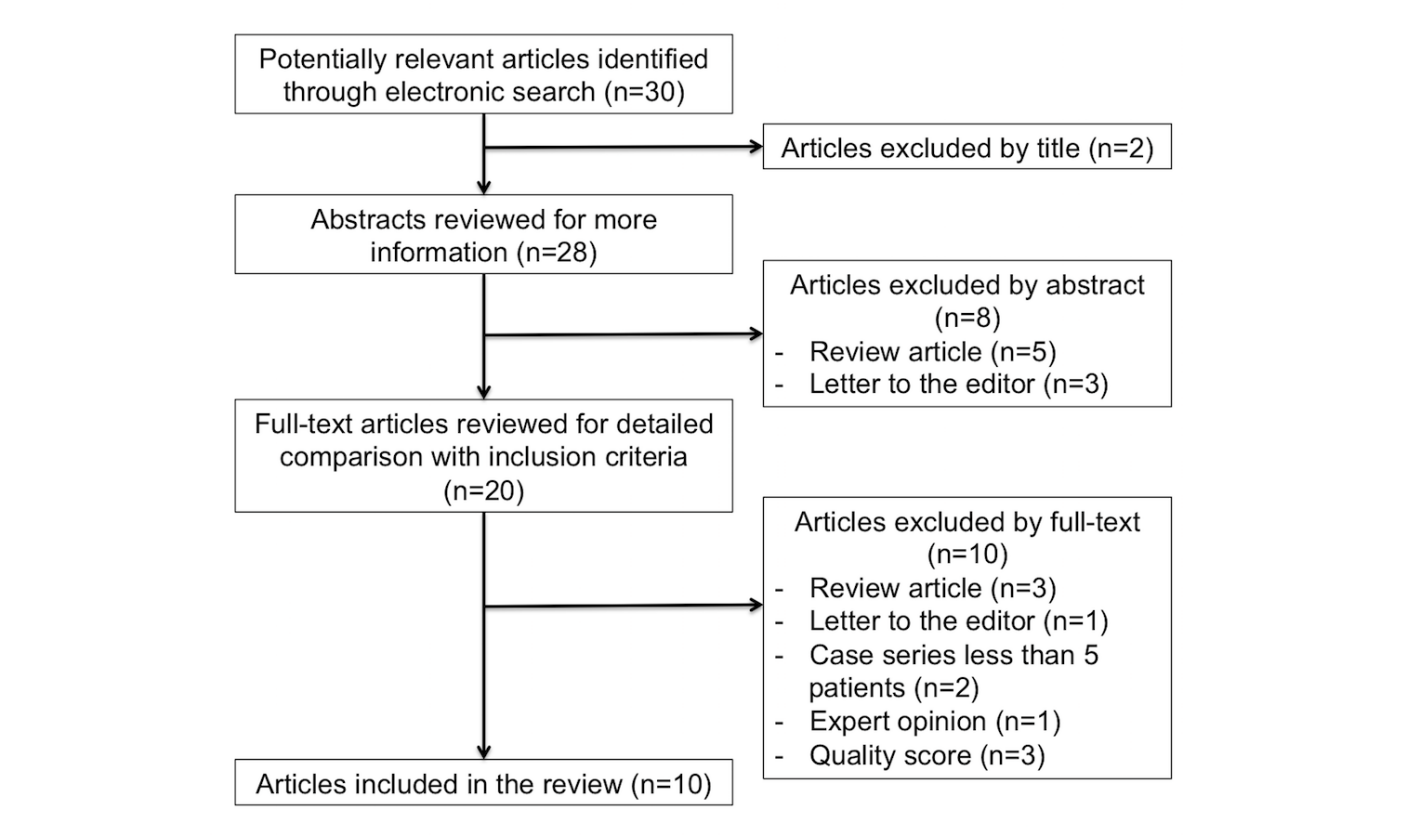
Study selection flowchart.

Using the search term “WhatsApp,” 30 articles were initially identified from the electronic databases consulted. After a detailed assessment of all references, followed by a screening process and a quality assessment (Figure 1), a total of 10 studies were determined to be eligible for inclusion in this systematic review.

**Table 1:** Summary of each study’s features and Oxford Levels of Evidence.

Authors	Year	Type of study	Level of evidence	Journal
Abanmy et al [15]	2012	Nonrandomized cohort study	III	Saudi Pharm J
Giordano et al [12]	2015	Individual cross-sectional study	II	Int J Med Infor
Khanna et al [16]	2015	Observational study	II	Eur J Orthop Surg Traumatol
Zotti et al [17]	2015	Randomized trial	II	Angle Orthod
Montag et al [5]	2015	Cohort study	III	BMC Res Notes
Willemse [18]	2015	Nonrandomized cohort study	III	Curationis
Vásquez-Silva et al [19]	2015	Nonrandomized cohort study	III	Rev Peru Med Exp Salud Publica
Muntaner-Mas et al [20]	2015	Observational study	II	J Sports Med Phys Fitness
Cheung et al [21]	2015	Randomized trial	II	J Med Internet Res
Dorwal et al [22]	2016	Nonrandomized cohort study	III	J Med Syst

According to the Oxford Levels of Evidence, there was no Level I study, though there were 5 Level II studies (1 individual diagnostic cross-sectional study with consistently applied reference standards and blinding, 2 randomized risk or benefit assessment trials, and 2 risk or benefit observational studies), and 5 Level III studies (1 prognostic cohort study, 1 treatment benefits nonrandomized controlled cohort study, and 3 screening nonrandomized cohort studies).

The summary of each study’s features and level of evidence is shown in Table 1.

### Basic Information About the Articles

All of the articles were originally written in English, with the exception of one article written in Spanish. Of the included studies, 2 were performed in India, and 1 was performed in each of the following countries: Brazil, China, Germany, Italy, Peru, Saudi Arabia, South Africa, and Spain. Five studies were conducted by medical professionals, 4 by other health professionals, and 1 by kinesthetic and sports professionals. Five studies investigated the applicability of the WhatsApp Messenger as either a communication tool between health care professionals or as a learning tool [12,16,19,21,22]. In the other 5 studies, the app was used either as a communication tool between health care professionals and the public or as a teaching tool to provide information on health care to the general population [5,15,17,18,20].

Among the studies conducted by medical professionals, 2 were performed by orthopedic surgeons, 2 were performed by general practitioners, and 1 was performed by pathologists. A brief summary of these papers is provided in Table 2.

**Table 2:** Summary of the studies conducted by medical professionals.

Authors	Specialty	Aim	Methods	Results	Conclusions
Giordano et al [12]	Orthopedics	To evaluate inter- and intraobserver agreement in the initial diagnosis and classification of images from tibial plateau fractures	Plain radiographs and CT^a^scans were obtained from 13 cases of tibial plateau fractures Images were photographed with a smartphone and sent to 6 observers via WhatsApp Messenger Observers were asked to determine standard deviation and type of injury, classification according to the Schatzker and the Luo classifications, and whether the CT scan changed the classification	Inter- and intraobserver agreement for both periods of the study ranged from excellent to perfect across all survey questions	Systematic use of the mobile app facilitated faster documentation and acquisition of the opinion of an experienced consultant when not on call
Khanna et al [16]	Orthopedics	To report the impact of the introduction of WhatsApp as an intradepartmental communication tool	25 consecutive admissions before and after the introduction of WhatsApp were included in the study 8 orthopedic residents answered 50 randomly arranged questions based on the 25 patients in each study period	Significant improvement observed in scores after introduction of WhatsApp	Introduction of WhatsApp as an intradepartmental communication tool can bring about improvements in patient-related awareness, in communication, and handovers among orthopedic residents
Vásquez-Silva et al [19]	Internal medicine	To assess the access, use, and preferences of information and communication technology by physicians who practice at a general hospital in Lima, Peru	A questionnaire explored the availability of ICT^b^, and physicians’ skills, time constraints, educational activities, and preferred search engines and technological applications, as well as their ICT preferences in education 211 physicians were surveyed	One in every 2 doctors use WhatsApp to exchange information and images and to perform interactive consultations within the hospital Different groups were formed according to specialty or department (cardiology, emergency, general practitioners, medical residents, etc)	Use of and access to ICT is common among doctors in this general hospital, and there is positive interest in its use in education
Cheung et al [21]	Internal medicine	To determine the effectiveness of WhatsApp and Facebook groups for preventing smoking relapses among quitters	Single-blinded, parallel, 3-arm pilot cluster randomized controlled trial allocating recent quitters, who had completed an 8-week treatment and reported abstinence for at least seven days Participants were allocated into a WhatsApp group (n=42), a Facebook group (n=40), and a control group (n=54) Two intervention groups participated in a 2-month Web-based group discussion with either WhatsApp or Facebook moderated by a trained smoking cessation counselor and received a self-help booklet on smoking cessation Control group only received the booklet	Fewer participants in the WhatsApp group reported relapse than the control group at the 2-month follow-up (OR^c^= 0.27, 95% CI 0.10-0.71) and 6-month follow-up (40.5%, 17/42 vs 61.1%, 33/54; OR=0.43, 95% CI 0.19-0.99); WhatsApp groups had more posts from moderator (median 60, IQR^d^25 vs median 32, IQR 7; *P*=.05) and from participants (median 35, IQR 50 vs median 6, IQR 9; *P*=.07) than their Facebook counterparts, but the difference was not significant	Intervention via the WhatsApp group was effective in reducing relapse probably because of enhanced discussion and social support Inactive discussion in the Facebook social group may have attributed to the lower effectiveness
Dorwal et al [22]	Pathology and Laboratory Medicine	To look at the impact of using the WhatsApp Messenger service in the laboratory management system by forming multiple groups of the various subsections of the laboratory	Thirty five members used this service for a period of 3 months, and their responses was measured on a scale of 1 to 10	Significant improvement in communication (the sharing of photographic evidence, information about accidents, critical alerts, duty rosters, academic activities, and getting directives from seniors) Some increase in the load of adding information to the application and disturbance in the routine activities, but the benefits far outweighed the minor hassles	Results suggest and reflect another communication revolution that will change the way information is shared in a health care sector, with hospital-specific apps

^a^CT: computerized tomography.

^b^ICT: information and communication technology.

^c^OR: odds ratio.

^d^IQR: interquartile range.

## Discussion

### Principal Findings

The development of smartphones has created new opportunities for integrating mobile technology into daily clinical practice in several fields of health care, mainly due to the ability to download and install custom apps [23]. Due to their portability, speed, simplicity, and ability to update, mobile apps are an ideal tool for quick reference and learning purposes or for communication between health professionals and the general public [23,24]. Due to these characteristics, mobile apps are currently one of the most commonly used tools for telemedicine worldwide.

WhatsApp is a recent technology startup founded to build a better short message service alternative [24]. Its use has increasingly drawn a wider range of interest as a communication and imaging chat system between health care professionals and patients, as well as among health care professionals themselves. In several regions around the world and particularly in rural areas and low- and middle-income countries, the use of WhatsApp has been shown to facilitate communication among health care professionals in terms of faster problem identification and immediate acute management [4,11,25]. Recently, studies have reported the increased presence of WhatsApp in medicine and many other health care fields, which reflects the increased acceptance of its use. This phenomenon is attributed to the fact that WhatsApp is a cost-effective, quick, reliable, and user-friendly tool. It is therefore able to provide a greater proportion of patients and the general public with guideline recommendations and treatment [9,12,13].

In this systematic review of the literature, we relied on a search for the term “WhatsApp.” The medical search was based on information from the standard MeSH medical ontology. Three different electronic databases were searched. They were chosen because they are high-quality representative Web pages written in plain English and provide good linkages between medical terminology and plain English words [26]. A total of 30 articles were initially identified, but after a detailed assessment of all of the references, a subsequent screening, and a quality assessment, 10 studies were determined to be eligible for inclusion [5,12,15-22]. Although our study demonstrates a positive role of WhatsApp usage for health care purposes, the studies found in the literature were found to be substandard in quality. In addition, there is a clear publication bias in the majority of the papers included in this review. The negative trial results were typically not reported by authors, which was the major reason for the publication bias and which was therefore one of the exclusion criteria. None of the studies were double blinded, and only one trial was single blinded. The remaining trials were either nonrandomized, observational, or cohort studies.

We analyzed and evaluated the quality of the studies by grading their level of evidence. Despite our findings of the utility of WhatsApp in the health care field, the level of evidence in each of the 10 studies included in this systematic review was relatively low. There were no level I studies, a finding which clearly demonstrates weaknesses and inconsistencies among the studies on this topic. Among the trials conducted by medical professionals, 3 were found to have a level II of evidence. The main reasons for this discrepancy were the lack of randomization, the irregular use of a methodology, and issues in data acquisition. In addition, the study PICO (acronym for P-problem, I-intervention, C-comparison, and O-outcome) did not match the question PICO in many of the trials. We know by far that in many instances, it is very difficult to frame a research question; however, a successful research project depends upon how well an investigator formulates this question based on the problems faced in day-to-day research activities and clinical practice [27].

Although issues of ethics and security were not discussed in any of the 10 trials included in this systematic review, new users of WhatsApp for medical and other health care–related purposes must consider cyber security and the ethical implications of telehealth. As stated by Gerard et al, when citing the American Medical Association’s Code of Medical Ethics, the information disclosed to a physician during the course of the patient-physician relationship is confidential to the utmost degree [28]. The incorporation of telemedicine as a clinical enterprise should be strongly consistent with ethical practices. The American Telemedicine Association has recently updated the policy with the caveat that telemedicine per se is “not a practice in and of itself” [29]. One of the greatest barriers to the use of telehealth is fear. For patients, this often means the fear of intrusion or becoming distanced from their provider. For providers, this fear is associated with the lack of security to prevent unauthorized access sensitive patient information. Being sensitive and responsive to these concerns, as well as to the human factors that impact mobile app use by patients and health care providers alike, will all be crucial for the present and future success of any telehealth system or app [30].

### Limitations

This paper presents several limitations. First, the authors studied only the WhatsApp Messenger app, although many other similar and popular messaging applications exist as social media. One of the major advantages of instant messaging for medical and other health care professionals include their capacity to identify, diagnose, and treat many life-threatening conditions in the acute scenario remotely. Preventing and minimizing the impact of noncommunicable diseases are some of the greatest challenges facing modern society [31]. In the actual study, we have tried to look at the impact of using the WhatsApp Messenger service for this purpose, as other studies have shown a higher effectiveness of this platform in terms of participation over other social media [21,23,32]. In reality, the use of WhatsApp Messenger can provide a solution to this by providing subtle structure to an erratic environment [32,33]. In addition, WhatsApp is seen to be a simple, cheap, and effective means of communication within the clinical health sector, and its use will grow [33,34].

Second, although we could find 10 papers eligible for inclusion in this systematic review, medical professionals conducted only 5 studies. Health professionals and kinesthetic and sports professionals conducted the other 5 studies. Therefore, the authors decided to make a brief summary of the medical papers. This study was a pragmatic evaluation of the impact of WhatsApp Messenger as an adjunctive tool for telemedicine, broadly defined as the “use of electronic information and communication technologies to provide and support health care when distance separates” the patient and the medical professional [1-3,35].

Third, a relatively small number of eligible studies were identified, and in general, they were very heterogeneous and presented a relatively low level of evidence, which reduces the trust that can be placed in their findings. These factors limited our ability to synthesize data and reach definitive conclusions. Maher et al identified the same problem in their systematic review of the effectiveness of online social network health behavior interventions [31]. As stated by these authors, “there is a possibility that studies with null findings have not been published and that the synthesis of data presented here gives an overly favorable account of effectiveness” [31,36].

### Conclusions

In conclusion, the pooled data presents compelling evidence that the mobile app known as WhatsApp Messenger is a promising system when used as a communication tool between health care professionals and the general public, as a method of communication among health care professionals themselves, or as a learning tool for providing information on health care to professionals or to the general population. However, high-quality and properly evaluated research is urgently needed, as are improvements in descriptions of the methodology and the study processes. These improvements will allow WhatsApp to be categorically defined as an effective telemedicine tool in many different fields of health care.
